# Theorising about child maltreatment: Narrative review on health education models, conceptual frameworks and the importance of the information and communication technologies

**DOI:** 10.3389/fpsyg.2022.841917

**Published:** 2022-08-02

**Authors:** Sagrario Gómez-Cantarino, Victoria Mazoteras-Pardo, José Rodríguez-Montejano, Cinzia Gradellini, Aliete Cunha-Oliveira, María Idoia Ugarte-Gurrutxaga

**Affiliations:** ^1^Faculty of Physiotherapy and Nursing, University of Castilla-La Mancha, Toledo, Spain; ^2^Research Group Nursing, Pain and Care (ENDOCU), University of Castilla-La Mancha (UCLM), Toledo, Spain; ^3^Health Sciences Research Unit: Nursing (UICISA: E), Coimbra School of Nursing (ESEnfC), Coimbra, Portugal; ^4^Azienda Unità Sanitaria Local-IRCCS of Reggio Emilia, University of Modena and Reggio Emilia, Modena, Italy

**Keywords:** nursing, abuse, children, education, theory, family, software, new technologies

## Abstract

Child maltreatment is conceived as a public health problem. Therefore, it is appropriate to analyse the explanatory models that deal with this behaviour, reflecting these postulates within the panorama of health education, which makes health professionals responsible for taking action. In order to do this, the theoretical context and the awareness of nursing students in relation to these theories must be analysed. In turn, the use of information and communication technologies in this field should be valued, due to their capacity to manage and systematise information, becoming a relevant tool when training future nursing professionals. Without forgetting that health informatics is a spectrum of multidisciplinary fields that includes the study of the design, development and application of computational techniques to improve healthcare. A review of the scientific literature was carried out, for which primary and secondary sources were consulted, tracing a search for data thanks to the keywords: ‘nursing’; ‘abuse’; ‘children’; ‘education’ and ‘theory’. During the second half of the 20th century, several health paradigms have been developed, which present different pathways to health education. There have also been three generations of theoretical models that attempt to analyse the public health problem of child maltreatment. This reflects the need for a transdisciplinary approach to child abuse, where there is no one explanatory model that is more appropriate than another, but where the choice of the health education paradigm and, within this, the most recommendable theory will depend on each situation.

## Introduction

The human mind represents a state of consciousness and subconsciousness that cannot be directly assessed or defined. The cognitive and subjective processes of people are individual, which make it possible to conceive the reality of each user in a unique way. One tool by means of which the deepest region of human psychology can be analysed is psychoanalysis (Papel et al., 2005; Poscheschnik, 2009), a research technique consolidated by the neurologist Sigmund Freud around 1896 (Alvarenga, 2006). This procedure allows for a possible interpretation of dream manifestations, longings, lack of self-esteem or free association of details (Tubert, 2000). The historian Roudinesco and Plon (2005, p. 1232) went further into Freud’s definition of psychoanalysis in 1922, determining that ‘Freud provided the most precise definition of the psychoanalytic framework by underlining that its theoretical “pillars” were the unconscious, the *Oedipus complex*, resistance, repression and sexuality (…)’ In turn, it is worth noting that the psychoanalysis proposed by Sigmund Freud constitutes one of the most important references for the understanding of the psychodynamic model, a conceptual framework that tries to provide a theoretical point of view on different elements, such as the human being and its development, or the proliferation of disorders, both physiological and psychological (Tubert, 2000; Roudinesco and Plon, 2005).

In the same way, these elements have been of special relevance in the field of education in Higher Studies, specifically in the Degree in Nursing, when forging a professional design of action and development of care. This requires an analysis of the complex reality of contemporary society, valuing its cultural aspects, social relations, the transmission of knowledge and the wide diversity of dynamic factors that make up the human being (Roudinesco and Plon, 2005), including information and communication technologies (ICTs). This factor, in relation to health sciences, has undergone multiple changes over time, even modifying the way of university teaching and health education. In fact, research in health informatics focuses on the applications of artificial intelligence in healthcare, generally centred in academic institutions. Therefore, the relationship between new technologies, health education and learning in health sciences is a relevant union in our days and, for this reason, it should be valued (Juanes Méndez, 2016). In order to develop such an interpretation of the prevailing reality, not only is the education provided at universities in the health sciences relevant, but also the self-realisation of the individual and the evolution of the inner world of students in this branch of health. This will allow a greater conception of human society by consolidating holistic and integral care, maintaining the perception that health is not something professional, but is based on the active and dynamic participation of people, with the aim of achieving the common good and individual improvements, with pre-eminence of the collective and social (Acuña González et al., 2014). This plays a relevant role when it comes to understanding Health Education (HE), understood as a set of intersectoral actions whose aim is to promote people’s well-being, a key function of health professionals, by providing the human community with the necessary competences for the promotion and protection of their comprehensive health (Riquelme Pérez, 2012).

Within the field of health education, the World Health Organization (WHO) has proposed three theoretical educational methods, which present different perspectives (Papel et al., 2005), with the aim of teaching and raising awareness among the population about different situations, including child abuse, which has accompanied humanity throughout its history (Suárez, 2001; López Jaramillo et al., 2013). This is one of the most heartbreaking and violent acts that can be practised on children and that are accepted for religious or disciplinary reasons (Suárez, 2001). These practices of physical, social and psychological affliction of children have even been used as educational and learning methods. In fact, in Roman society, it was considered that the ‘pater famili’ abandoned, sold or even killed their children (Cunyas Chalco, 2019). It was not until the Middle Ages that infants were recognised as human beings, but with the conception of the child as a corrupt being, who must be socialised, redeemed through discipline and punishment (Fry, 1993; Enesco, 2009).

In the second half of the 19th century, publications focused on child abuse began to appear, describing its impact, especially at the physiological level, as demonstrated in 1860 by the French physician Auguste Ambroise Tardieu (1818–1879), who came to describe quite particular injuries in paediatric cases. Almost a century later, in 1946, the radiologist John Caffev enunciated the first formal concepts on child abuse, when publishing studies on multiple bone fractures in infants, coming to value another relevant detail: the incoherent explanation that parents of abused children can provide (Cunyas Chalco, 2019). In the 1970s, the first conceptual frameworks on child maltreatment emerged, in order to understand, firstly, the functioning of these acts based on the supposed presence of psychiatric disorders in the parents or guardians of the children. As the twentieth century progressed, more subtle theoretical variants, both sociological and socio-environmental (Gil, 1970), proliferated in the search for an understanding of child abuse.

**Figure 1:**
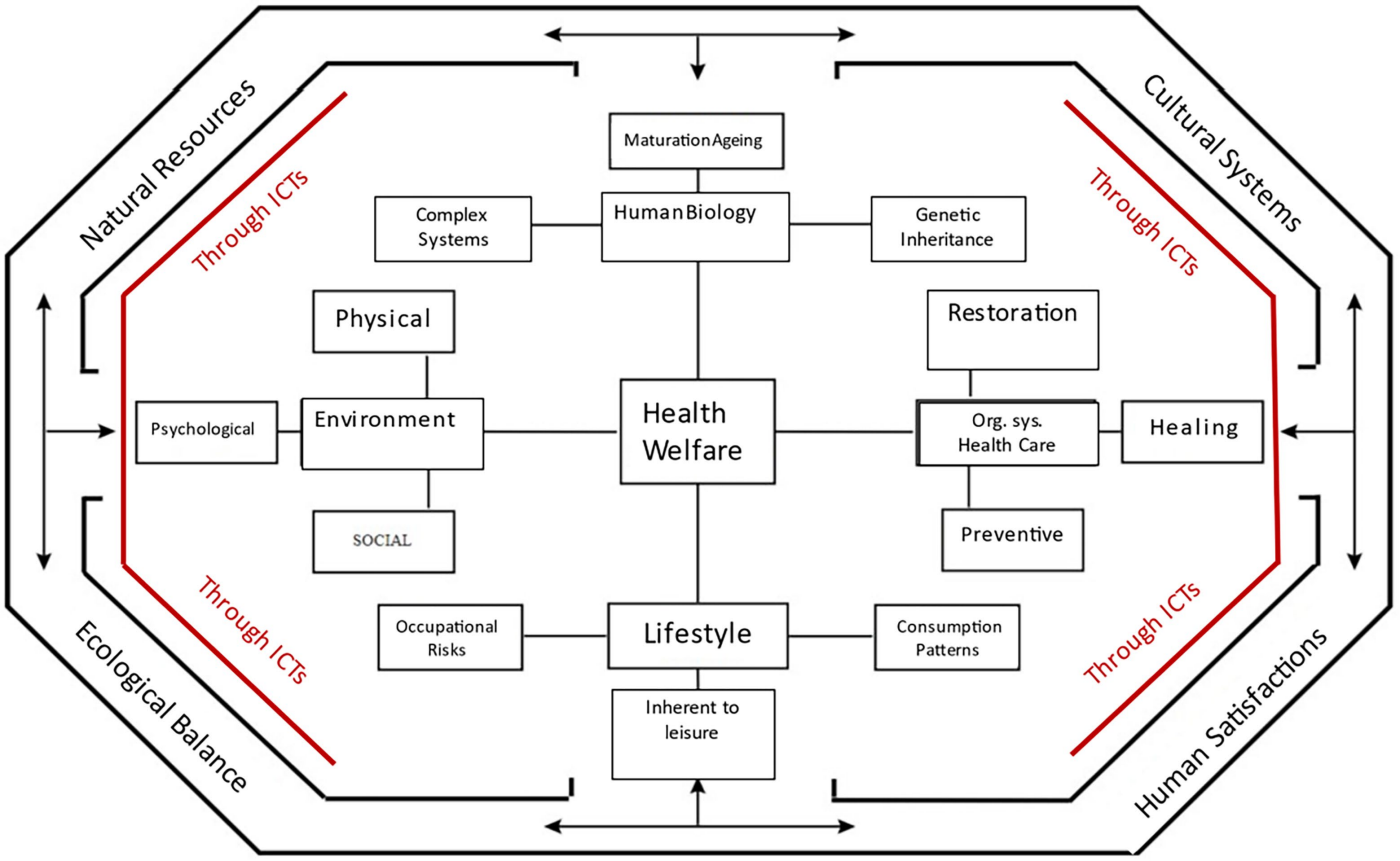
Epidemiological model for health field analysis in health promotion, preservation, and recovery. Modified by the author from: Dever (1976), Blum (1981), and Coutino et al. (1991).

In relation to these parameters, the general aim of this study is to describe the conceptual frameworks on child maltreatment according to the explanatory models of HE proposed by the Pan American Health Organization (PAHO) and the World Health Organization (WHO) by comparatively analysing the different theoretical frameworks (PAHO/WHO, 1996), in addition to identifying relevant insights into concrete forms of intervention in nursing education and practice for the promotion, maintenance or restoration of health in accordance with child maltreatment (Figure 1).

The study will also represent a significant shift in existing research by examining the use of various explanatory models as an efficient tool for nursing practice and ultimately for the various health professions. This study is a very timely contribution to the international analysis of the teaching of health education, as well as child maltreatment, in Higher Education programmes and specifically in nursing. The University of Castilla-la-Mancha (Toledo, Spain), the University of Reggio Emilia (Italy) and the University of Coimbra (Portugal) form the collaborative network of this research.

## Materials and methods

### Study design

This research aims to show a detailed, selective and critical study that integrates the essential information from a unitary and overall perspective (Icart et al., 1994). It is an exploratory study of analytical-descriptive approach with a qualitative approach in a multisectoral context, with great utility in education, as well as related fields (Day, 2005) which was developed from January to June 2021.

This narrative review has been carried out using five stages of action taking into account the qualitative data of the various explanatory models to be addressed. The initial stage of a review study is to identify an observed problem in healthcare and propose an objective (Peters et al., 2015); in this case, the overall purpose of this review was to describe publications with social content in relation to child maltreatment and the educational measures to be imparted in the human community to discard them. Therefore, it is intended to provide a better understanding of the phenomenon to be studied, since for nursing the greatest potential is represented in the development of clinical practice, as it allows for the creation and review of scientific evidence (International Council of Nurses, 2014; Peters et al., 2015; Knoester and Plikuhn, 2016).

### Search strategy

In order to carry out this review, a series of phases of action were established, bearing in mind the qualitative data of the various explanatory models to be addressed. In the first phase, a bibliographic search was carried out in the following electronic databases: PubMed, Science Direct, CINAHL (Cumulated Index of Nursing and Allied Health Literature), Scopus, Cuiden, as well as Google Scholar (Table 1).

**Table 1:** Search strategy in databases.

**Database**	**Search Strategy**	**Limits**	**Filters**
PubMed	[(nursing) AND (care)] AND [(child) OR (health) AND (society) AND (culture)] AND [(education) OR (new technologies)]	Title Article English/ Spanish/Portuguese	24 items filtered
Science Direct			19 items filtered
CINHAL			17 items filtered
Scopus			12 items filtered
Cuiden			18 items filtered
Google Scholar			35 items filtered

Own elaboration of the authors.

**Figure 2:**
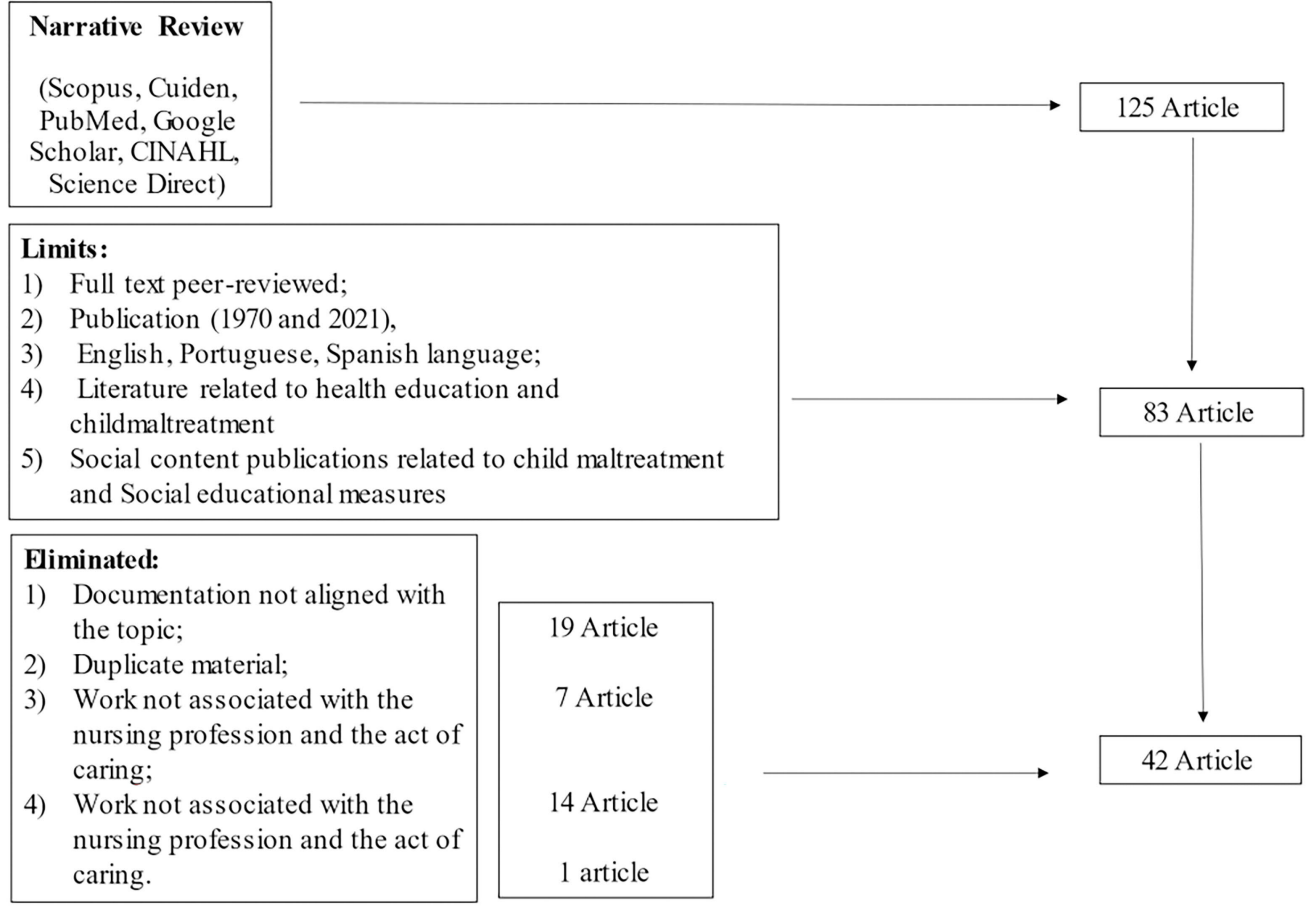
Article filtering strategy (Author’s own elaboration).

The papers retrieved during the searches were checked against the following inclusion criteria (1) original full-text report published in a peer-reviewed journal; (2) publication period between 1970 and 2021, in order to review the literature in reduced time; (3) manuscripts in Spanish, English and Portuguese language; (4) documentation related to conceptual frameworks of health education in line with child maltreatment, and (5) publications with social content related to child maltreatment and educational measures to be provided in the human community. Exclusion criteria were (1) documentation not aligned with the topic; (2) duplicate material and (3) papers aligned with the study topic, but not associated with the nursing profession and the act of caring. In this phase, 125 articles related to the topic were selected, but 42 that met the established inclusion criteria were finally reviewed and studied in depth (Figure 2).

In a second phase, documentation in paper format was reviewed in the Library of the University of Castilla-La Mancha (Toledo Campus), in the Public Library of Castilla-La Mancha, in the archive of the Faculty of Medicine of the Complutense University of Madrid, as well as in the Municipal Archive of Toledo. The discussion focused on several sub-themes: (1) history of children’s rights; (2) history of child abuse; (3) child, family and health and (4) ethical abuse and nursing. A pre-analysis of the material found was carried out. In addition, the reading of books and chapters was carried out, which have been cited throughout the development of the study. There were 20 books, of which 12 books and 5 chapters were studied in depth in order to address the objective of the study.

In the third phase, a manual and/or electronic review was carried out of official documents extracted from the International Council of Nurses and the Coordination of Community Health, the Directorate of Medical Benefits of the Mexican Institute of Social Security, as well as the Ministry of Health, Social Services and Education of the Spanish Autonomous Communities. A total of 8 documents of this type of bibliography were reviewed, but 3 of them were studied in depth.

### Data analysis

The bibliography used for this review was subjected to an inferential interpretation by the researchers. An attempt was made to understand the already researched and written reality related to the models of child maltreatment linked to the educational framework of both PAHO and WHO, confronting it with the social reality regarding violence towards infants. This involved the establishment of analysis through integrated categories within child maltreatment models organised into methods: (1) didactic or prescriptive; (2) awareness raising and (3) participatory with a focus on human development. These thematic methods, which are not mutually exclusive, serve as a basis for understanding the unmet needs or demands that constitute a problem for the human population.

After applying the inclusion and exclusion criteria, a total of 56 documents were selected for analysis.

## Results

Every human being is immersed in a context or suprasystem that influences, to some extent, his or her health-illness continuum. Social, environmental or physiological factors or aspects influence the thoughts, feelings and actions of the person, independently of the conscious vision of the subject (García, 1998). Likewise, the child’s natural and artificial environment, which can influence his or her psychological and affective development, should also be taken into account. Being surrounded by architectural barriers, without free spaces or playgrounds, can aggravate the psyche of the abused infant, interfering with his or her integral well-being, causing emotional damage that can come to symbolize indelible marks for the whole life of the person (Ramírez Calixto and Cedeño Sandoya, 2018). And it has been evidenced that child abuse contributes to a high degree of emotional maladjustment on the part of minors, a fact that leads to the need, on many occasions, for a reevaluation and cognitive-emotional attention (Weissman et al., 2019; Bonet et al., 2020). This is fundamental since an incorrect emotional regulation strategy can lead to psychopathologies (Kim et al., 2021), including self-injurious behaviours (Guérin-Marion et al., 2020). Therefore, in this section, which concerns a common good of human society such as the state of health, nursing care emerges as a public service and an opportunity for the population (Miguélez-Chamorro and Ferrer-Arnedo, 2014). The care provided by nursing staff functions as a frontier or transition between population levels, sectors and health services (Cuevas et al., 2008), always seeking to act as a coach, facilitator, companion or educator (Miguélez-Chamorro and Ferrer-Arnedo, 2014).

Thus, nursing, and more specifically, community nursing, is highly linked to HE, which fosters the consolidation of self-care competencies by the individual and at the family level (SITEAL, 2018; Soto et al., 2018). This includes the basic functions of health promotion and education, disease prevention, care of sick or disabled people, rehabilitation and reintegration of individuals into the social framework. In this regard, the International Council of Nurses in the United States that the responsibilities of nurses include ‘promotion of a safe environment, research, participation in health policy formulation and management of health systems and patients, and education’ (Shamian, 2014). Therefore, when educating the human community, explanatory models must be taken into account that serve as a basis for shaping the process of action in the face of unmet needs or demands that constitute a problem for the human population.

**Figure 3:**
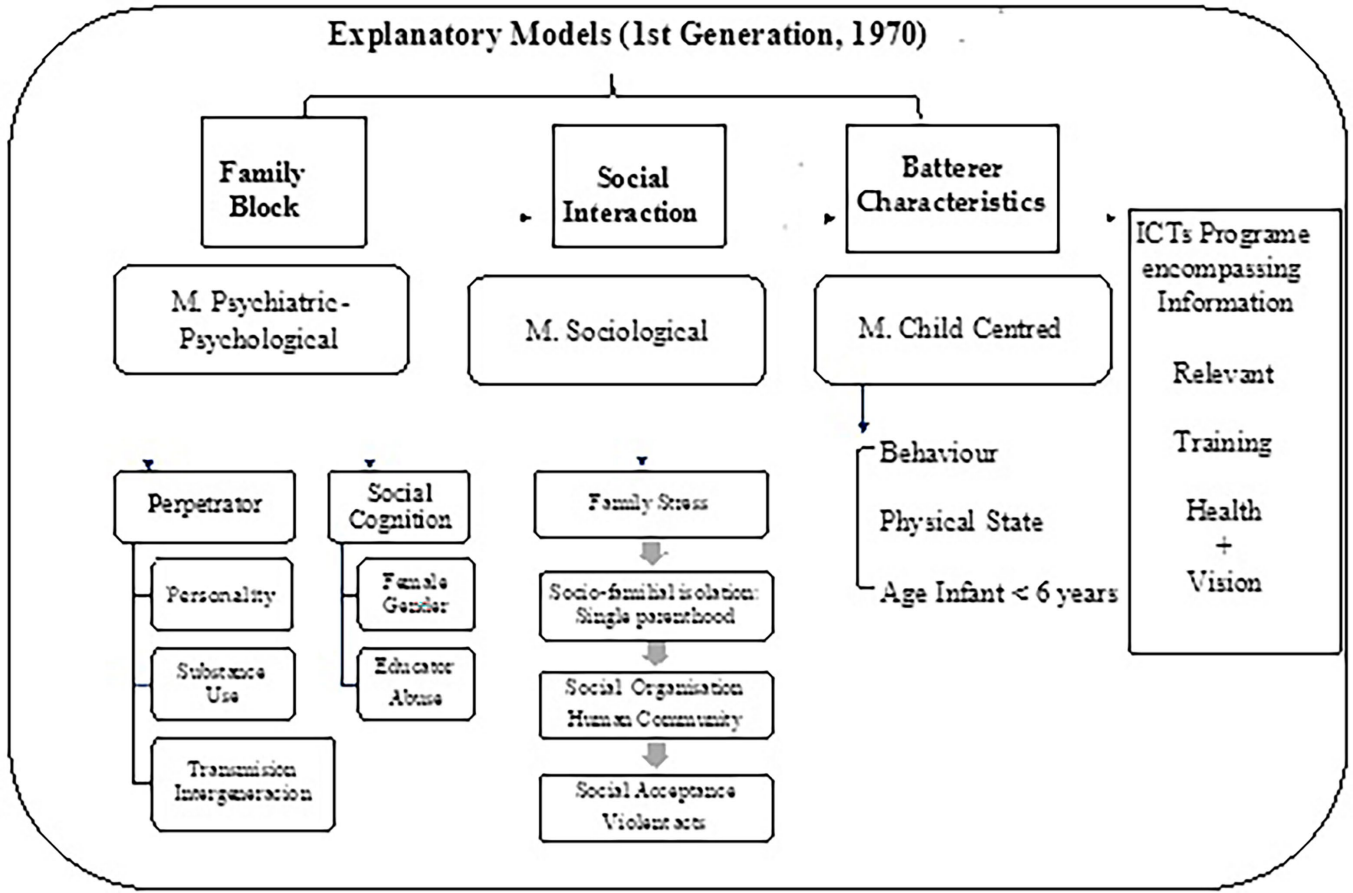
First-generation explanatory models. Health education and learning in health sciences converge with the development of new information technologies: a relevant link between training and health care (Author’s own elaboration).

Within the framework of health education itself, PAHO/WHO offers three methods with different approaches, which are not mutually exclusive (Gil, 1970): (1) the didactic or prescriptive method; (2) the awareness-raising method and (3) the participatory method focused on human development. The prescriptive method, also called the first-generation method, is a model that is framed in the development of an approach aimed at the detailed exposition of goal-oriented contents, without valuing the subject’s demand. A dissemination of information is carried out in which a classical pedagogy is applied, generating the sole guilt of the patient, while a prescription or detailed message is elaborated (Fernández Oliva et al., 2004), without feedback to the professional who imparts this model. The awareness-raising (or second generation) method focuses on an integrative pedagogy, in which the subject plays an active and dynamic role, encouraged by the educator. In this case, such analysis is based on the premise of studying multidimensional causes, delving into social, environmental or psychological conditions, thanks to the feedback generated by the people. These kinds of models function as systems that generate self-confidence and progressive independence on the part of individuals, with the ultimate goal of social development (Lois, 2013). As sociologist Everett M. Rogers (1931–2004) put it, such progress would be ‘a broadly participatory process of social change, which seeks to expose social and material advances (including equality, freedom, and other valued aspects) to the majority of the population through gaining control over their own environment’ (Rogers, 1976). Finally, it is worth mentioning the third-generation models or participatory methods centred on human development (such as the co-orientation model), which present a different perspective on health education in that it is not the health professionals who initiate the educational approach, but the human community that possesses the capacity and self-sufficiency necessary to make its own decisions, thus influencing its management and state of community health. The nursing profession is a participant in this process, not a key figure, as the basic protagonist is the population itself (Figure 3).

These conceptual frameworks have evolved over time, thanks to the changes in the health paradigm offered by speculative contributions such as the theory of health beliefs or the theory of self-efficacy, postulated by Bandura (1986), Mora and Araujo (2008). The same applies to the theoretical analysis of child maltreatment, which is described by the WHO as a computation of the abuse and neglect of children under 18 years of age. It encompasses the various types of physical, psychological or sexual abuse that may threaten or cause harm to the health of children or violate their rights (Arrieta Vergara et al., 2014). Within this scope, it would include what is done (action), omission and what is done inappropriately, i.e., negligence (Doria Martínez and Navarro Chong, 2016). Likewise, at present, the transmission of knowledge, using computer technologies, virtual resources and simulation systems, constitutes one of the great technical-scientific revolutions (Juanes Méndez, 2016), having special relevance in the health sciences, contributing multiple dimensions to the educational process (Ochoa Vásquez, 2009). And, therefore, they can be used as teaching tools on child abuse, both in their theoretical aspects and in the ability to diagnose and prevent possible cases, especially new variants of child abuse such as cyberbullying or sexting. In this sense, it is essential to integrate ICT into the university teaching environment, through computer programmes that systematise the information on the area to be dealt with, in this case, child abuse.

However, this poses a huge challenge for teachers, who have to reinvent themselves and know how to manage these new models of education, channelling information effectively in order to achieve, as effectively or more effectively, the necessary competences in health science subjects. The educational disciplines involved in teaching-learning combine transversal, specific and general competences with informatics fields such as data science, information technology and behavioural informatics (Juanes Méndez, 2016). According to the researcher Pérez López: ‘This will be the only way to be able to adequately process the incessant avalanche of information […]; on the other hand, it is also the only way to avoid irrational consumerism, i.e., that which causes the most striking or easy to assimilate to end up being the chosen or most credible thing” (Pérez López, 2006, p. 928).

### First-generation models of child maltreatment

From the historical context, the explanatory models of child maltreatment are encompassed in various aspects. The first perspective focuses on the family block, exposing as the aetiology of child abuse an altered family dynamism between the child and the parents or guardians, without reasoning in a more profuse aetiology. The second perspective of these theoretical frameworks studies the social interaction at the origin of child abuse. While the third wave of explanatory models delves into the characteristics of the abuser, delving into the intrapsychic of the perpetrator (Manso, 2006) when looking for the explanation of why maltreatment is generated.

In this way, the first strand flourished in the 1970s, through various theoretical frameworks. On the one hand, it is worth highlighting the psychiatric-psychological model, which tries to explain child abuse from a psychopathological point of view of the parents or legal guardians. Within this framework, different correlations are proposed between child maltreatment and the perpetrator, mainly through the perpetrator’s personality, which may be configured by depression, low self-esteem and inability to self-control impulses or anxiety (Zuravin, 1988; Culp et al., 1989; Manso, 2006). Similarly, there are several studies that outline this perspective, stating the use of toxic substances (see drugs) by the legal guardian as a trigger for the act of maltreatment of the paediatric case. Also, it may be an intergenerational transmission, where abused subjects later act as parents who abuse their children (Zuravin, 1988; Milner, 1995). Within the psychiatric-psychological model, social cognition must also be assessed, given the fact that studies suggest the difficulties of reflexivity and emotional recognition that parents, usually mothers, may experience (Kropp and Haynes, 1987), leading to distorted expectations of what is expected of children (Soto et al., 2018), giving rise to some type of child maltreatment as a rectification or educational measure.

Another model that proliferated in the 1970s, apart from the psychiatric-psychological one, was the sociological model, which not only encompasses psychological aspects, but also social variables. Furthermore, it indicates how family environment conditions and cultural elements are key factors in the triggering of child abuse (Chaffin et al., 1996; Cantarino et al., 2016). This model encompasses four key aspects to be studied: (1) family stress; (2) the social isolation of the family; (3) the social organisation of the human community and (4) the social acceptance of violent acts (Riquelme Pérez, 2012). In relation to the social isolation of the family block, it is necessary to assess whether there is a low socio-economic level (Hillson and Kuiper, 1994). This fact causes a stigma at the level of the human community, influencing the type of social and family interactions (Zuravin, 1989) and fostering the likelihood of abuse or mistreatment of children. This situation occurs as a form of emotional and cognitive relief for the parents, a fact that is even more pronounced in the case of a single-parent family, mainly generated by a separation of the couple (Sack et al., 1985).

**Figure 4:**
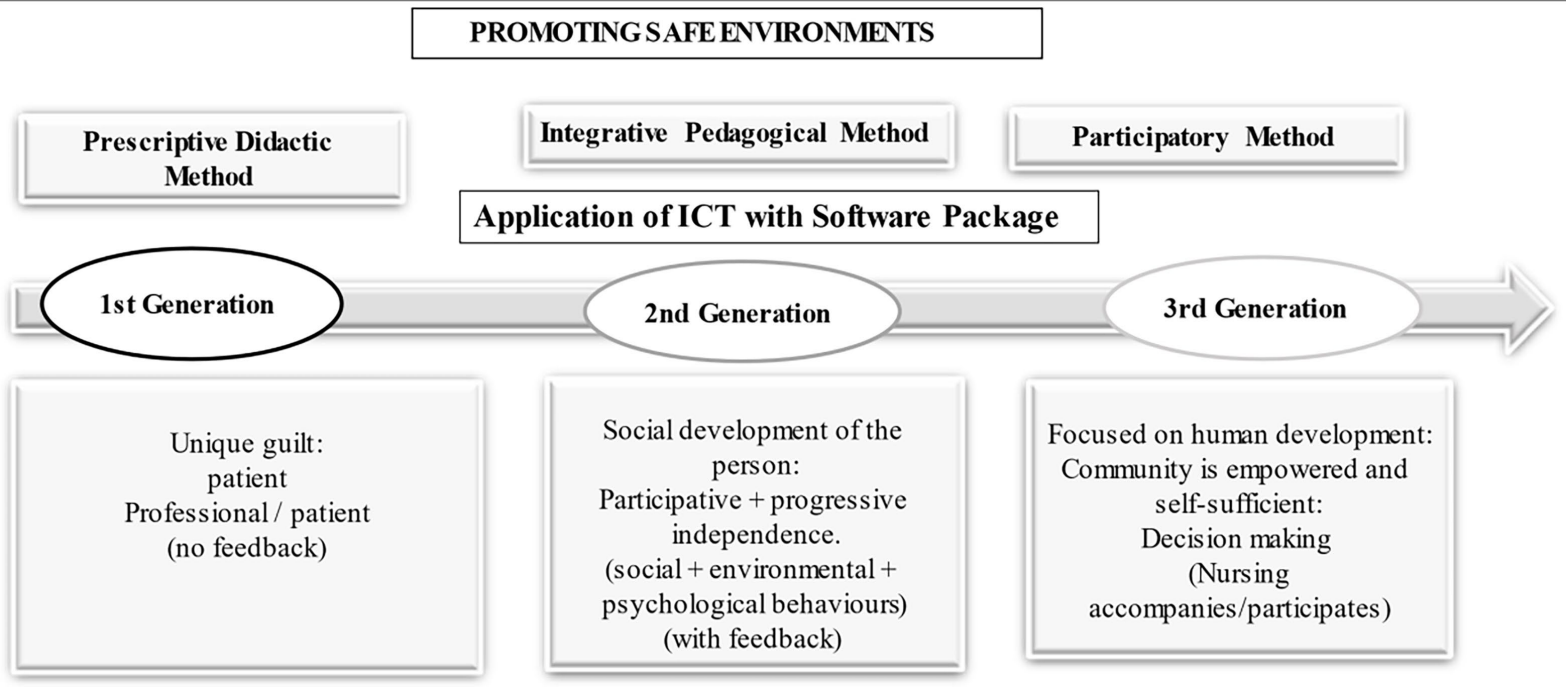
Health education method: application of ICT with software package to improve the training of future professionals and health and social care (Author’s own elaboration).

The last of the traditional explanatory methods of child maltreatment is child-centred, considering that the child can exhibit certain characteristics that make it aversive for parents or legal guardians (Azar, 1991; Figure 4; Table 2).

**Table 2:** Dcumentation included within the scope of child maltreatment.

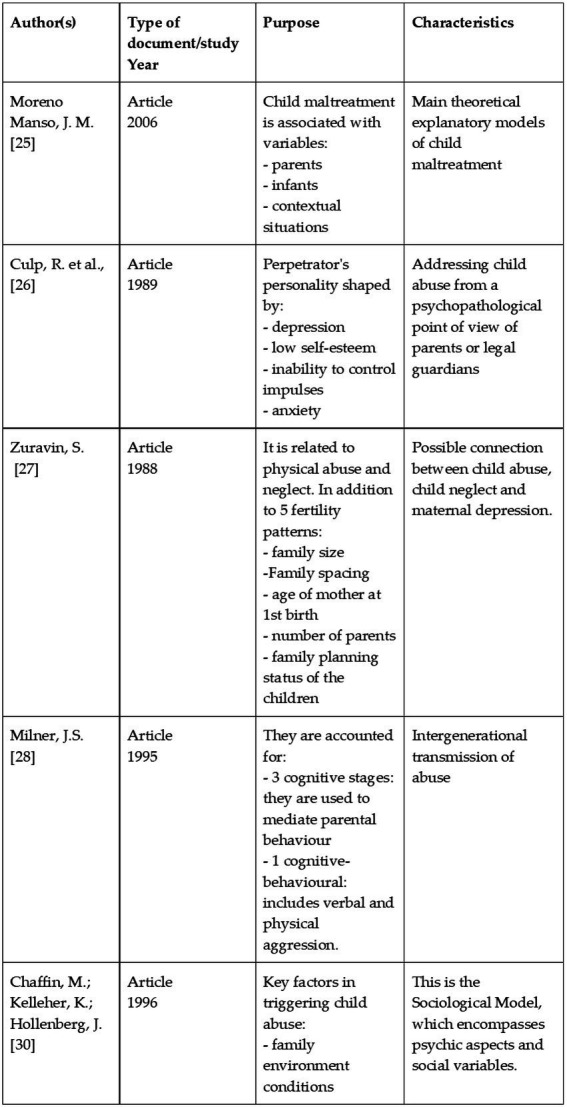

Source: Own elaboration of the authors.

### Second-generation models of child maltreatment

On the other hand, it was in the 1980s when a new perspective began to proliferate in relation to the causality of child maltreatment, deriving in a different current to that developed in previous years. In this way, the social interaction approach emerged, which conceives that child maltreatment is due to a variety of variables, both parents, the infants and the contextual situations that arise. Therefore, these second-generation models are more complex than first-generation theoretical-explanatory frameworks (Riquelme Pérez, 2012). Within this theoretical current, several frameworks are worth highlighting: firstly, Belsky’s ecological model, a system of four interacting levels of interdependent systems, which allows for the linking of parents (their behavioural and psychological characteristics), the family block (microsystem), the human community (exosystem) and culture (macrosystem), taking into account the environments in which the infant interacts. With this theory, Belsky seeks to establish the fact that a specific human society is an organised whole, in which according to the elements that sustain it, these can come to influence the family nuclei, in sensory, perceptual and cognitive aspects such as violence, education or discipline (Riquelme Pérez, 2012).

Another of the second-generation conceptual frameworks to be assessed is the transactional model of Cicchetti and Rizley (1981), a theory focused on the multi-causality of child maltreatment, which is the consequence of an imbalance between enhancing factors (increasing the probability of child maltreatment) and dampening factors (decreasing the risk of child abuse). Enhancers can be of various kinds, such as biological (see physical malformations that strain the parent–child relationship), psychological (presence of parental mental illness), historical (history of abuse) and ecological (inappropriate environment for family harmony). Thus, child maltreatment would be caused when the enhancing factors predominate over the buffering factors (Cicchetti and Rizley, 1981).

A year after the publication of Cicchetti and Rizley’s theoretical framework, a new explanatory model appeared that derives from the school of behavioural psychology (Riquelme Pérez, 2012), which acts as an experimental philosophy of analysis of human behaviour (Skinner and Ardilla, 1975). Thus, Vasta's (1982) two-component model posits the existence of two components or forces that will influence the triggering of child maltreatment. These are the use of punishment as a means of disciplining children, and the emotional hyper-reactivity of parents. The linking of these two elements can lead to compulsive acts of violence against children, which are perceived as forms of teaching within the family nucleus (Vasta, 1982). Finally, this theoretical current will end in 1987 with Wolfe’s transitional model, which derives from Vasta’s postulates, by exposing a three-stage sequencing of child maltreatment, where four components influence, in this case, the evolution of maltreatment, the psychological processes associated with anger, the potentiating factors and the protective factors against maltreatment (Vasta, 1982; Table 3).

**Table 3:** Documentation included within the scope of child maltreatment.

**Author(s)**	**Type of document/study** **year**	**Purpose**	**Characteristics**
Cicchetti, D.; Rizley, R. [36]	Article 1981	Aetiology of intergenerational transmission and sequelae of child maltreatment.	Child maltreatment is caused when enablers predominate over buffers.
Skinner, B. [37]	Book 1974	Explanatory model: behaviourist school	Experimental philosophy of human behaviour analysis
Vasta, R. [38]	Article 1982	The option of using: - punishment: to discipline the child. - infant: punishment to create discipline. - parents: emotional hyper-reactivity.	It posits the existence of components or forces that will influence the triggering of child maltreatment.
Wolfe, D. [39]	Book 1987	A sequence of child maltreatment is presented in which it influences: - evolution of maltreatment - psychological processes - factors that promote maltreatment - protective factors of maltreatment	Infant involvement in psychopathology acts.

Source: Own elaboration of the authors.

### Third-generation models of child maltreatment

In the 1990s, a new stream of explanatory models developed, which seek to find the aetiological cause of why child maltreatment is triggered, from a psychological perspective, thus replacing the descriptive and not very explanatory view of the second-generation models (Riquelme Pérez, 2012). This third generation will consist of two main theoretical frameworks, both Milner’s theory of social information processing and Hillson and Kuiper’s stress and coping theory.

Hillson and Kuiper's (1994) stress and coping theory focuses on how each individual person copes with anxiety, and how anxiety can lead to physical and behavioural impairment. Thus, attention is focused on the possible anxiogenic stimuli that influence the abuser, on the cognitive evaluations that the abuser makes of his or her situation with the child and the way he or she copes with it. Assessing whether the behaviour is positive or maladaptive for the pediatric case can generate a situation that leads to an act of child abuse (Hillson and Kuiper, 1994; Riquelme Pérez, 2012). In turn, Milner's (1995) theory of social information processing exposes the existence of cognitive errors that influence the parent’s thought pattern, causing an altered judgement and a schema of misinterpretation of situations to be present.

Especially in terms of the conception of their children’s behaviours, which generates distorted integration of perceptual information that leads to the execution of a response, based on the production of child abuse (Milner, 1995; Riquelme Pérez, 2012).

## Discussion

After analysing the history of the explanatory models of health education and the conception of child abuse (according to a possible aetiological cause), a correlation can be seen in terms of the paradigmatic change in the study and explanation of different aspects, such as the human being, its development, health and the existence of affections, in this case, abuse in paediatric cases.

For this reason, a comparative relationship is proposed between the different theoretical frameworks, which allows a relevant vision of the concrete ways of intervening in education and nursing practice, for the promotion, maintenance or restoration of health in cases of child abuse. Thus, taking the three non-exclusive focal methods (Culp et al., 1989) of PAHO/WHO as a basis, the following overview of evolution and theoretical designation is given:

The prescriptive method focused on a transmission of information, in which the sole culpability lies with the patient (based on the professional-subject relationship of Hippocratic medicine; Entralgo, 1970; Gracia, 2002; Guadarrama and Campos, 2020), with the main determinants of illness being biological factors. In this case, neither social interaction nor cultural or economic influence is taken into account, but rather an imposition of factors and values. Traditional explanatory methods of child maltreatment can be accommodated within this theoretical line, because links between child maltreatment and the personality of the maltreater are exposed. See the consumption of toxic substances, altered expectations of what is expected of children (Riquelme Pérez, 2012), and even an intergenerational transmission of the experiences of maltreatment (Milner, 1995). Thus, in this first correlation, the first-generation model of health education encompasses the exposition of the psychiatric-psychological theoretical framework, the sociological model and the one centred on the paediatric case, all of them explanatory models where the prestige and coherence of biomedical science prevails (Moreno et al., 1995; Gómez-Cantarino et al., 2020).

At the same time second-generation models in health education were developed in the 1960s, reflecting a cultural anthropology approach, allowing a deeper understanding of the subjectivity of each subject, thus achieving a more contextualised approach (Moreno et al., 1995). Thus, the intrinsic characteristics of the person and those consolidated by contact with society are valued. From this new line of health education, models based on behaviourist postulates will be forged that allow progress towards new approaches, such as ecological ones (Colom Cañellas, 2004; Hernández-Girón et al., 2012), thanks to the configuration of a precise technology or methodology. When addressing the explanatory models of child maltreatment, it can be seen how the second-generation models of health education are the basis for the consolidation of the theoretical frameworks of social interaction on child maltreatment developed in the 1980s (see Belsky’s ecological model or Cicchetti and Rizley’s transactional hypothesis).

Similarly, there is a clear transition between second and third-generation models with respect to child abuse, a fact that can be explained by a paradigm shift in health. This would justify the existence of Milner’s theory of social information processing and the stress and coping postulates of Hillson and Kuiper. Models developed during the 1990s, when the “eco-epidemiological” paradigm was consolidated, which analyses the health problem through multiple determinants, belonging to various spheres, not only biomedical, but also psychological and social (Hernández-Girón et al., 2012). This emerging health paradigm can enable both a specific therapeutic approach (at the individual level) and a preventive approach (at the population level; Hernández-Girón et al., 2012; Hernández et al., 2017). Therefore, the existence of this new vision of understanding a public health problem has made it possible to conceive child maltreatment from a broader and more holistic theoretical perspective (Table 4).

**Table 4:** Correlation of health education models and theories of child maltreatment.

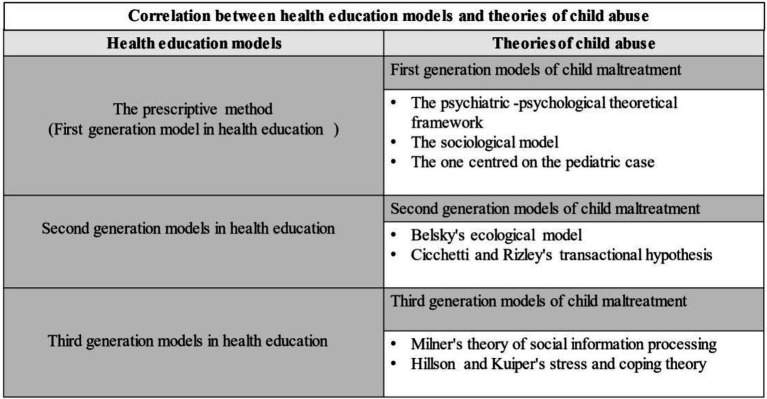

At the same time, it should be noted that there are insufficient elements to determine which of the explanatory model or paradigm is the correct one. This will depend, in part, on the approach to each case of child maltreatment and the competencies displayed by the health professionals, especially those belonging to the nursing discipline when addressing the problem under study (Hernández-Girón et al., 2012). This situation highlights the need to raise awareness and teach higher education students, who are studying nursing, about the various existing explanatory models and the paradigmatic changes in health knowledge that exist, so that the complex reality of each case of child maltreatment can be taken into account (Roudinesco and Plon, 2005). It is here where the use of ICTs as a systematic mechanism of university teaching should be used, facilitating simple, attractive and dynamic learning that allows the necessary competences to be obtained in this field, and even to promote and generate future specific computer programmes on child abuse within direct user care. Studies carried out (Arredondo, 1993; Hernández-Girón et al., 2012; Priegue Caamaño and Cambeiro Lourido, 2016) reflect the responsibility of education professionals in influencing the future competences of the new generation of professionals. Therefore, this aspect becomes even more important when it comes to health issues. Despite this, there is often a lack of knowledge and adequate university training on child abuse, both within and outside the family (Soto et al., 2018). Although it is true that this situation is usually solved with new future lines of research focused on this panorama, in order to be able to tackle a serious public health problem such as child maltreatment (Gonzalez et al., 2021).

## Conclusion

After analysing the general approach of this study, some reflections are raised. From the professional perspective, it is emphasised that teachers must be prepared and responsible for training future generations of nursing professionals to acquire the minimum competences to prevent and deal with a situation of child maltreatment. This can be presented in a theoretical as well as a practical way. The theory provides the basic building blocks that guarantee the implementation or follow-up of a process. This foundation can be corroborated with the Nursing Care Process (NCP), developed by the nurse theorist Linda Hall (1906–1969) in 1955. With this theoretical framework, it is possible to show the correct and precise sequences for action by any nursing professional in the face of a health condition, allowing the work carried out to be evaluated and studied. Despite this, at present, the theoretical efforts of nursing professionals to develop knowledge through theories and models have not been successfully implemented in clinical practice, considering that this is based on the performance of routine procedures. However, such care acts have a clear logic, as they are derived from theoretical postulates.

Thus, through practice in the different rotation devices, both in primary care and specialised care, as well as in secondary education centers, it is a good time to develop the competences acquired on a theoretical level on child abuse, considering this training to be more focused on higher courses within the degree and postgraduate nursing degree.

Likewise, it is exposed that the evolution of the theoretical postulates on child abuse is correlated with the educational theoretical methods proposed by the WHO, which establish different perspectives of teaching and awareness of the population on multiple public health problems, bearing in mind cultural patterns that influence the sense, evolution and thought of human society. This demonstrates the need for a transdisciplinary approach to child maltreatment, where there is no one explanatory model more suitable than another, but the choice of the health education paradigm and, within this, the most recommendable child abuse theory will depend on each situation. It is also important to highlight the importance of valuing the use of information and communication technologies (ICT) in this field, due to their high capacity to manage and systematise information, making them a relevant tool for the correct training of future health professionals, especially in the case of child abuse, as it is a public health problem. In the words of researchers Nuria Fabrellas and Jordi Galimany ‘Health professionals themselves should promote and consume the use of these applications, as we are witnessing a democratisation of health information that can facilitate health promotion and disease prevention’ (Fabrellas Padres and Galimany Masclans, 2019, p. 7).

## Author contributions

SG-C, AC-O, and JR-M contributed to the conception and design of the study. SG-C, AC-O, JR-M, CG, and MU-G performed the data collection, organisation, and analysis. SG-C and VM-P wrote the first draft of the manuscript and sections of the manuscript. All authors contributed to the article and approved the submitted version.

## Funding

This research was funded by the European Regional Development Fund (ERDF), Own Research Plan. Resolution of 19/01/2021 (DOCM 27/01/2021), 2021-GRIN-31242. University of Castilla-La Mancha (UCLM).

## Conflict of interest

The authors declare that the research was conducted in the absence of any commercial or financial relationship that could be construed as a potential conflict of interest.

## Publisher’s note

All claims expressed in this article are solely those of the authors and do not necessarily represent those of their affiliated organizations, or those of the publisher, the editors and the reviewers. Any product that may be evaluated in this article, or claim that may be made by its manufacturer, is not guaranteed or endorsed by the publisher.

## Abbreviations

ICT: Information and Communication Technologies
WHO: World Health Organization
PAHO: Pan American Health Organization
HE: Health Education
